# The Role of Bioactive Compounds in Human Health and Disease

**DOI:** 10.3390/nu17071170

**Published:** 2025-03-28

**Authors:** Jacqueline I. Alvarez-Leite

**Affiliations:** Departamento of Biochemistry and Immubology, Federal University of Minas Gerais, Belo Horizonte 31270-901, MG, Brazil; jalvarezleite@gmail.com

## 1. Introduction

Bioactive compounds, natural chemicals found in foods, herbs, and other natural sources, have attracted significant attention for their potential to influence health and fight disease. Unlike macro- and micronutrients necessary for metabolism and human nutrition, bioactive compounds offer benefits that can improve overall well-being and prevent or mitigate various health conditions.

Bioactive compounds have several biological activities. They exert effects such as antioxidants, anti-inflammatories, antimicrobials, and immunomodulator agents [1]. These properties have made these compounds a focus of interest in health and disease prevention [2]. Polyphenols, found in fruits and vegetables, act as antioxidants with therapeutic or adjuvant potential in therapies for diseases such as obesity, diabetes, cardiovascular disease, and cancer, among others [3]. Compounds such as resveratrol, found in grapes and red wine, have shown promising anticancer properties by inhibiting the growth and proliferation of cancer cells [4,5]. Bioactive peptides derived from proteins can modulate blood pressure and glucose metabolism, offering benefits for controlling hypertension and diabetes [6,7]. In addition, numerous phytochemicals in spices and herbs have been identified as possessing anti-obesity and lipid-lowering effects, contributing to weight management and metabolic health [7,8].

This Special Issue of *Nutrients* delves into the multifaceted effects of bioactive compounds, showcasing groundbreaking research and analysis that explores their potential in combating various health challenges. Below is an overview of the articles contained in this Special Issue.

### 1.1. Anti-Obesity Potential of Pleurotus ferulae

Hong et al. (contribution 1) investigate the anti-obesity effects of *Pleurotus ferulae*, a mushroom used mainly in Asian cuisine. The authors showed that *P. ferulae* extracts reduced weight gain and adiposity in obese mice fed high-fat diets. *P. ferulae* extract also reduced serum lipid, improved profiles, and impaired adipocyte differentiation, suggesting its potential to prevent and control obesity.

### 1.2. Pediatric Perspective on Bioactive Compounds

Bioactive compounds are not well studied in pediatric populations. In this regard, Cecchi et al. (contribution 2) presented a systematic narrative review that evaluated the literature concerning bioactive compounds present in supplements from a pediatric standpoint. They focused on types, sources, bioavailability, physiological effects, and clinical implications. Several bioactives reinforce the immune system, reducing the incidence and severity of infections in children. They could also support optimal growth trajectories and cognitive function. By functioning as prebiotics, these compounds could improve digestion, enhance nutrient absorption, and improve overall gastrointestinal health. However, clinical trials are necessary to establish the safety and efficacy of bioactives in pediatric populations. Ensuring the quality, purity, and consistency of bioactive-enriched supplements is crucial. While current evidence is promising, more research is needed to confirm their long-term benefits and potential risks.

### 1.3. Resveratrol: Pharmacological Properties and Challenges

Farhan and Rizvi (contribution 3) present a summary of recent research on the pharmacological properties of resveratrol, known mainly for its potent antioxidant activity. Resveratrol has shown anticancer capabilities by blocking all stages of carcinogenesis. It can also benefit other non-communicable diseases such as diabetes and cardiovascular disease. Clinical and experimental studies suggest that moderate resveratrol supplementation could benefit health. However, there is a long pathway before total knowledge of its action mechanism and bioavailability is achieved, so further research is required before indicating it as a therapy for the prevention of disease.

### 1.4. Capsaicin and Non-Communicable Diseases

Silva et al. (contribution 4) present a comprehensive examination of capsaicin, the spicy component of chili peppers, in the treatment of non-communicable diseases such as obesity, diabetes, and dyslipidemia. Recent studies highlight the benefits of capsaicin, including improved antioxidant status and gut health. Although research on the use of capsaicinoids and capsinoids as adjuvants for treating obesity, diabetes, dyslipidemia, and cancer is growing, extensive studies remain limited. Capsaicin is shown to improve antioxidant and anti-inflammatory status, induce thermogenesis, and reduce white adipose tissue while also affecting food intake and intestinal health. Despite promising clinical results, most studies are limited to under 12 weeks, and additional investigations are needed to determine optimal dosage, treatment duration, and long-term safety. The impact of capsaicin on gut microbiota and its role in cancer prevention is still in the early stages, requiring further exploration to clarify whether capsaicin is protective or promoting in cancer cases.

### 1.5. Health-Promoting Effects of Chroogomphus rutilus

Han, Luo, and Xu (contribution 5) revised the chemical compositions and health-promoting effects of *Chroogomphus rutilus*, a wild edible mushroom. Rich in vitamins, proteins, minerals, polysaccharides, and phenolics, C. rutilus exhibits antioxidant, antitumor, immunomodulatory, antifatigue, hypoglycemic, gastroprotective, hypolipidic, and neuronal protective properties that could be beneficial as an adjuvant in the treatment of several diseases. However, the mechanism driving such actions has been poorly studied. Studies addressing *C. rutilus* actions are still restricted to cellular or animal experiments. Further clinical research and practice are needed to verify its safety and efficacy and to promote its use in clinical practice. Despite the remarkable pharmacological effects of *C. rutilus* in vitro and in vivo, there is a lack of information on its safe use, therapeutic index, and risk–benefit ratio in humans. These data will be essential for the development of new pharmaceutical preparations based on the active principles of *C. rutilus*.

### 1.6. Health Benefits of Anthocyanin-Rich Vaccinium Species

Kopystecka et al. (contribution 6) explore the health benefits of anthocyanin-rich *Vaccinium uliginosum* and *Vaccinium myrtillus,* known as bilberry. These berries exhibit antioxidant, anti-inflammatory, anti-cancer, and apoptosis-reducing activities. The authors believe that they could be helpful as dietary supplements for preventing cancer diseases and cataracts or as a part of sunscreen preparations. Clinical studies confirm their anti-inflammatory, anticancer, and apoptosis-reducing activities, making them valuable additions to a healthy diet. Although studies suggest the prevention and possible treatment of cancer with *V. uliginosum* blueberries, their benefits are still unconfirmed and more clinical research is needed.

### 1.7. Yacon Flour and Colorectal Cancer

Grancieri et al.’s original study (contribution 7) investigated the effects of yacon flour on inflammation and colorectal cancer in an animal model. They showed that yacon flour reduces inflammation in mice with induced colorectal cancer, indicating its potential as a dietary intervention. The treatment with yacon flour increased fecal secretory immunoglobulin A levels and decreased lipopolysaccharides, tumor necrosis factor-alpha, and interleukin-12. Although yacon flow ingestion did not influence oxidative stress, it reduced inflammation by increasing fecal secretory immunoglobulin A levels and decreasing lipopolysaccharides, tumor necrosis factor-alpha, and interleukin-12. These results make yacon flour a promising food for reducing the damage caused by colorectal cancer. Nonetheless, clinical studies are necessary to confirm its action and safety in those patients.

### 1.8. Chlorella Macronutrient Bioactivity

Lorenzo et al. (contribution 8) review the bioactivity of the macronutrients of Chlorella, a marine microalga rich in protein and containing all the essential amino acids. The macronutrients in Chlorella have been shown to improve exercise performance and reduce fatigue, attributed to its antioxidant, anti-inflammatory, and metabolic activities. The authors claim that Chlorella proteins increase intramuscular free amino acids and their utilization, sparing glycogen and improving the fatty acid β-oxidation capacity. The cell wall of Chlorella possesses carbohydrates that help maintain a gut microbiota balance and diversity. The authors also state that alpha-linolenic and linolenic fatty acids can contribute to physical performance by favorably modifying the fluidity of cell membranes. This makes Chlorella a promising dietary supplement for exercise-related nutrition. However, as it occurs with several bioactive compounds, more scientific and metabolic studies are necessary to confirm its benefits for humans.

### 1.9. Mexican Medicinal Plants in Glucose Homeostasis and Weight Management

Torres-Vanda and Gutiérrez-Aguilar (contribution 9), in their systematic review, explore the use of Mexican medicinal plants such as *Momordica charantia* L., *Cucurbita ficifolia bouché*, *Coriandrum sativum* L., *Persea americana* Mill., and *Bidens pilosa* in managing glucose homeostasis and controlling body weight. It has been observed that aqueous and organic extracts from these plants can have hypoglycemic effects by increasing insulin secretion and sensitivity, improving pancreatic β cell function and glucose tolerance, and regulating body weight. It is assumed that these effects are due to the different compounds, such as saponins, phenolic compounds, antioxidants, tannins, triterpenes, avocation B, cytoglobin, etc. Besides the analysis of the action mechanism of each of these plant-derived compounds, there are few studies of their toxicologic potential. In this way, it is still not possible to recommend doses for their hypoglycemic and body weight control effect. Therefore, it is necessary to study the different extracts using in vivo and in vitro models to validate their implications for glucose homeostasis and body weight control.

### 1.10. Bioactive Compounds and Breast Cancer

Breast cancer remains a major cause of death worldwide. This review (contribution 10) highlights the potential effects of polyunsaturated fatty acids and other bioactive compounds in terms of inhibiting molecular and signaling pathways associated with breast cancer. This review offers valuable insights into how these dietary components may provide natural strategies for the prevention of breast cancer and treatment. The authors conclude that Bromelain, ω-3 PUFAs, sulforaphane, and indole-3-carbinol are promising adjuvants for traditional treatment and chemoprevention for breast cancer due to the low risk of side effects and toxicity. However, using dietary compounds and phytochemicals as part of standard treatment for BC requires further investigation.

## 2. Conclusions

In summary, bioactive compounds play a key role in improving health and fighting diseases, mainly due to their anti-inflammatory and antioxidant properties. Their ability to modulate physiological processes and target specific disease pathways makes them a focal point in nutritional and medical research. As our understanding of these compounds deepens, their applications in preventive health care and therapeutic interventions continue to expand, offering promising avenues for improving human health and well-being.
